# Is There a Role of Using a Rapid Finger Prick Antibody Test in Screening for Celiac Disease in Children?

**DOI:** 10.1155/2019/4504679

**Published:** 2019-10-07

**Authors:** Kristina Baraba Dekanić, Ivona Butorac Ahel, Lucija Ružman, Jasmina Dolinšek, Jernej Dolinšek, Goran Palčevski

**Affiliations:** ^1^Department of Pediatrics, University Hospital Center Rijeka, Istarska 43, 51 000 Rijeka, Croatia; ^2^Office of Project Development-Project Office, Municipality of Maribor, Ulica Heroja Staneta 1, Slovenia; ^3^Department of Pediatrics, University Medical Center Maribor, Ljubljanska 5, 2 000 Maribor, Slovenia

## Abstract

**Introduction:**

Celiac disease (CD) is an autoimmune disease triggered by gluten in genetically predisposed individuals. Despite the increasing prevalence of CD, many patients remain undiagnosed. Standard serology tests are expensive and invasive, so several point-of-care tests (POC) for CD have been developed. We aimed to determine the prevalence of CD in first-grade pupils in Primorje-Gorski Kotar County, Croatia, using a POC test.

**Methods:**

A Biocard celiac test that detects IgA antibodies to tissue transglutaminase in whole blood was used to screen for celiac disease in healthy first-grade children born in 2011 and 2012 who consumed gluten without restrictions.

**Results:**

1478 children were tested, and none of them were tested positive with a rapid test. In 10 children (0,6%), IgA deficiency has been suspected; only 4 of them agreed to be tested further for total IgA, anti-tTG, and anti-DGP antibodies. IgA deficiency was confirmed in 3 patients, and in all 4 children, CD has been excluded.

**Conclusion:**

Our results have not confirmed the usefulness of the POC test in screening the general population of first-grade schoolchildren. Further research is needed to establish the true epidemiology of CD in Primorje-Gorski Kotar County and to confirm the value of the rapid test in comparison with standard antibody CD testing.

## 1. Introduction

Celiac disease (CD) is an autoimmune disease involving innate and adaptive immune responses triggered by the gluten ingestion in genetically predisposed individuals. In persons with HLA-DQ2 and/or DQ8 haplotypes, activated immune reaction results in small intestinal mucosal damage with villous atrophy, crypt hyperplasia, and an increased number of intraepithelial lymphocytes [1, 2]. Patients with CD may present with gastrointestinal symptoms; however, a substantial number of patients present with atypical extraintestinal symptoms of variable severity [2–4].

CD is a common disorder with the overall prevalence of 1%, with differences among countries (Germany 0.3%, Italy 0.7%, Finland 2.4%, and USA 1%) [1, 2, 5]. The prevalence substantially increased in the last 50 years [6]. However, the majority of patients are not identified; the data shows that almost 90% of patients, both children and adults, remain undiagnosed, possibly because of the high proportion of asymptomatic or oligosymptomatic patients [2, 3, 7]. After the last quarter of the 20th century, the dramatic shift from typical gastrointestinal manifestations to atypical and asymptomatic presentations has been noticed [2, 8].

There are few data about the incidence and prevalence of CD in Croatia. Only limited data from 10-year research from limited region exist; the cumulative incidence is 1.9 : 1000 life-births and prevalence 1 : 461 [9, 10].

Patients with CD have a modestly increased risk of malignancy and mortality [11]. Untreated illness is associated with numerous long-term complications, for example, delayed puberty, other autoimmune disorders (thyroid disease and diabetes mellitus), cerebellar ataxia, epilepsy, neuropsychiatric disorders, infertility, osteoporosis, small-for-date births, and malignancies (enteropathy-associated T cell lymphoma, small intestinal adenocarcinoma) [3, 4, 8, 12]. There is a strong evidence that undiagnosed CD is associated with nearly 4-fold increase risk of death compared to people without it [6].

Strict adherence to gluten-free diet (GFD) reduces the rate of morbidity and mortality [8], emphasizing the importance of early detection of patients who benefit from GFD [4].

Specific subgroups of individuals have an increased risk for CD, among these are first-degree relatives of CD patients and people with other autoimmune diseases (type 1 diabetes mellitus, autoimmune thyroiditis, and autoimmune hepatitis) and specific genetic disorders (Down syndrome, Turner syndrome, Williams syndrome, and IgA deficiency) [2]. Current ESPGHAN guidelines recommend active search for CD among these subgroups [13].

Despite the low rate of diagnosis, there are still no general recommendations for screening in the general population [1]. Based on the research of Greco et al., the burden of unrecognized CD patients will grow substantially in the Mediterranean region with an estimated number of 5 million cases in 2020; the estimated medical costs caused by delayed CD diagnosis are about €4 billion during a 10-year period. This emphasizes the need for simplified diagnostic protocols that will be available not only in specialized centers but also in rural areas [14]. Highly sensitive and specific point-of-care tests (POCT) might be a solution to shorten diagnostic delays. Besides, the data clearly shows that the mass screening could be the best strategy for secondary CD prevention [8]. There are few studies that examined the role of CD screening in Europe based on the increased prevalence of the disease [15–17].

Conventional laboratory methods (anti-transglutaminase 2 (tTG) IgA and anti-endomysial (EMA) autoantibodies) are expensive, not easily available, and difficult to use for mass screening [3, 4]. Therefore, rapid methods of antibody detection using blood from finger pricks that can be performed at the point of care have already showed their efficacy [3, 4, 18, 19].

The aim of our study was to determine the frequency of CD among first-grade schoolchildren in Primorje-Gorski Kotar County, Croatia, using a rapid point-of-care test.

## 2. Materials and Methods

### 2.1. Subjects

We screened first-grade schoolchildren from elementary schools in Primorje-Gorski Kotar County, Croatia. All children attending the first grade born in 2011 and 2012 were eligible for the study. Children already diagnosed with CD on GFD and children without CD, but who do not consume gluten for other reasons (e.g., allergic to gluten or wheat and parents' decision for not eating gluten), were excluded from the study, as well as children who have already been tested for CD during the last year. The goal of the study and principles of testing were presented to parents in every school, and written informed consent was obtained. Only children with signed informed consent were included in the study.

The team consisting of three pediatricians and two trained nurses visited all schools. The screening period lasted for 6 months, from September 2018 to February 2019. The study was a part of the Focus IN CD project (CE-111) cofinanced by the EU Interreg Central Europe Program and was approved by the Ethics Committee of the University Hospital Center Rijeka and Croatian Ministry of Science, Croatia.

### 2.2. Screening Procedure

The Biocard Celiac Test, Ani Biotech, Vantaa, Finland, was used for screening. This test is based on endogenous tissue transglutaminase (tTG) found in the erythrocytes of patients. According to the manufacturer's instructions, 10 *μ*l of whole blood is drawn and instilled into the 0,5 ml buffer, which causes hemolysis. As a consequence, tTG is released from erythrocytes. Three drops of the hemolyzed blood are added to the application field on the test. Persons with CD have circulating IgA anti-tTG specific antibodies that bind to released tTG. These complexes can bind to the solid surface coated with tTG-capturing proteins and anti-IgA antibodies labelled with a colloidal gold particle. As a result, in the case of CD, a visible test line is formed. A control line serves as a proof that the blood sample and the reagents moved over the test line. The results can be interpreted after 5 minutes, but no longer than 10 minutes; positive results can be seen already after 1-2 minutes. The test is negative if there is only line in the control area and positive if there are visible lines in both test and control areas, and in case of IgA deficiency, there are no lines in any of the areas. The sensitivity and specificity of the test were shown to be different in different age groups; in younger than 16 years, the sensitivity was 99% and specificity was 97%, while in older than 16 years, the sensitivity was 93% and specificity was 97% [19, 20].

Children with eventual positive results or the ones with suspected IgA deficiency were referred to Clinical Hospital Center Rijeka for total IgA measurements, IgA anti-tTG measurements (IDS, automated chemiluminescence immunoassay, CLIA) with a cutoff value of 7 U/ml, and IgG anti-DGP measurements (IDS, automated chemiluminescence immunoassay, CLIA) with a cutoff value of 7 U/ml.

## 3. Results

Primorje-Gorski Kotar County is located in the western part of Croatia, and Rijeka is the capital city. According to the census in 2011, it has 296195 inhabitants. There are 60 elementary schools in the county with a total of 2391 children in the first grade in the school year 2018/2019.

There was a total number of 1893 children whose parents attended parent meetings and agreed with the participation of their children in the study. According to inclusion criteria, children with known CD (*n* = 2) and children who already had CD testing within one year (*n* = 35) were excluded from the screening. Parents of 258 children refused to participate, and 120 children were absent from school on the day of the screening because of other reasons (e.g., illness).

**Figure 1:**
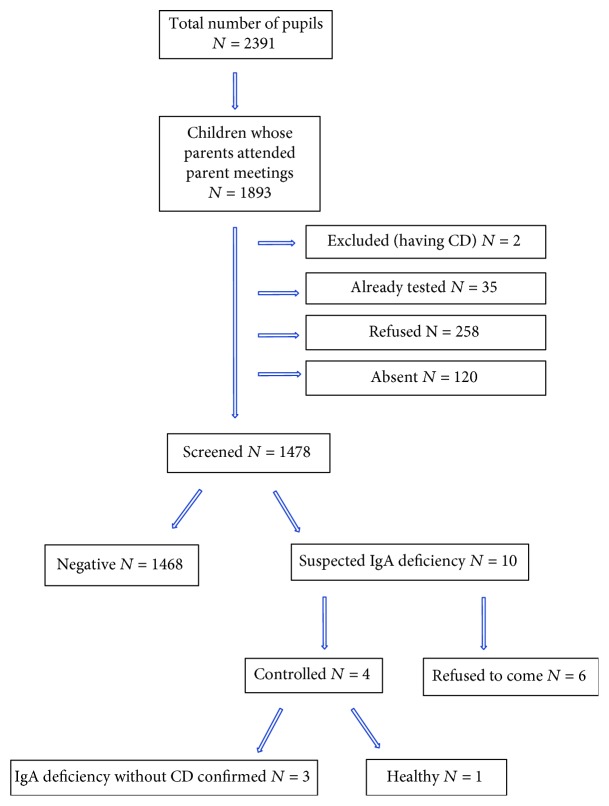
Results of the screening.

We screened 1478 children (61.82% of all eligible children). There were 964 (65.22%) girls and 514 (34.78%) boys. There was no invalid test reported. We did not find any patients with a positive rapid test, and 10 children were suspected to have IgA deficiency. They were referred to the Department of Pediatrics in Clinical Hospital Center Rijeka for total IgA, anti-tTG, and anti-DGP antibody measurements. Out of 10 children, only 4 of them came for the testing. IgA deficiency was confirmed in 3 patients who all had low IgG anti-DGP antibodies, and in one child, the total IgA level was normal and CD-specific antibodies were low (Figure 1). The data with IgA and CD-specific antibody levels is listed in Table 1.

## 4. Discussion

CD is one of the most frequent genetically based diseases of humankind [21], and majority of patients are misdiagnosed or not diagnosed at all [3, 7, 21]. There is ongoing discussion whether to screen for CD and whom to screen [22]. The World Health Organization (WHO) provides criteria for mass screening [7, 23, 24]: the disease must be common; screening tests must be simple, fast, and accurate and acceptable in different cultures; early clinical detection should be difficult; treatment must be available; and undiagnosed disease can lead to severe complications. CD clearly fits majority of the criteria. There are some open questions that need further investigations, including the degree of the risk for severe complications in asymptomatic individuals [20], cost-benefit ratio of the screening, benefit and compliance of GFD in asymptomatic individuals, and the appropriate age when to perform the screening [7, 8, 24, 25]. Generally, screening must be performed early enough to prevent late complications of the disease, but since a proportion of patients develop the disease later in life, early screening could miss them [7, 8, 26].

Nowadays, there are still no recommendation for mass screening [8, 21, 24, 25, 27]. According to ESPGHAN guidelines, screening should be undertaken for high-risk groups [13].

Our study on 1478 first-grade children tried to establish the prevalence of CD among 7-year-old children, the age by which a significant proportion of CD should have developed, and the potential use of rapid CD testing in general population screening. If the screening method is simple, the cost-benefit balance could be favorable even though benefits are only moderate [3].

Rapid POCT is cheap and easy to perform in comparison to standard CD testing [3, 4]. Studies made with the Biocard celiac disease test kit showed sensitivity, specificity, and positive and negative predictive values comparable with a standard CD test (anti-tTG and EMA), all higher than 93% [3, 19]; it was successfully used in screening first-degree relatives of CD patients, but the study was conducted on a small number of subjects [4], and in determining prevalence of CD among school-aged children in Turkey [18]. Although Comba et al. [18] had a representative sample, the lack of their study was the possibility of missing IgA-deficient patients with CD.

We were not able to detect children with CD. There are several possible explanations. First, prevalence among countries differs significantly; it ranges from 0.3% in Germany to 0.7% in Italy 0.7%, 1% in USA, and 2.4% in Finland [1, 2, 5]. According to our best knowledge, there are no data on CD epidemiology in Croatia, so our data could reflect lower prevalence of CD in Croatia compared with other countries. Second, we found 10 children with possible IgA deficiency and higher risk for CD development. Only four children came to our hospital to control IgA, anti-tTG, and anti-DGP levels. They were all negative for CD-specific antibodies, but there is a possibility that among the other 6 children whose parents refused to come for the specific CD antibody testing there are ones with CD. Third, although we followed the producer's instructions completely, there is still possibility of unintentional mistake. The test is qualitative in its nature, so a slightly visible test line could be missed.

## 5. Conclusion

To conclude, based on our study results, the POC test was not shown to be a useful tool in mass screening. Further research is needed to establish the incidence and prevalence of CD in Croatia, with more data to confirm the value of rapid tests in comparison with standard antibody CD testing.

## Figures and Tables

**Table 1:** Levels of total IgA, IgA anti-tTG, and IgG anti-DGP in children with suspected IgA deficiency after the rapid test.

Initials	Gender	Total IgA	IgA anti-tTG (U/ml)	IgG anti-DGP (U/ml)
LK	F	1.6	2	1.1
AB	M	<0.4	<0.8	<0.8
JT	M	0.3	1.1	1.6
MK	M	0.2	0.9	2.7

## Data Availability

The patients' personal data used to support the findings of this study are restricted by the Ethics Committee of the University Hospital Center Rijeka in order to protect patients' privacy. The general data of results of rapid tests used to support the findings of this study are available from the corresponding author upon request.

## References

[1] Green P. H. R., Lebwohl B., Greywoode R. (2015). Celiac disease. *The Journal of Allergy and Clinical Immunology*.

[2] Guandalini S. (2017). The approach to celiac disease in children. *International Journal of Pediatrics and Adolescent Medicine*.

[3] Korponay-Szabó I. R., Szabados K., Pusztai J. (2007). Population screening for coeliac disease in primary care by district nurses using a rapid antibody test: diagnostic accuracy and feasibility study. *BMJ*.

[4] Popp A., Jinga M., Jurcut C. (2013). Fingertip rapid point-of-care test in adult case-finding in coeliac disease. *BMC Gastroenterology*.

[5] Mustalahti K., Catassi C., Reunanen A. (2010). The prevalence of celiac disease in Europe: results of a centralized international mass screening project. *Annals of Medicine*.

[6] Rubio– T.,. A., Kyle R. A., Kaplan E. L. (2009). Increased prevalence and mortality in undiagnosed celiac disease. *Gastroenterology*.

[7] Ravikumara M., Nootigattu V. K., Sandhu B. K. (2007). Ninety percent of celiac disease is being missed. *Journal of Pediatric Gastroenterology and Nutrition*.

[8] Meijer C., Shamir R., Szajewska H., Mearin L. (2018). Celiac disease prevention. *Frontiers in Pediatrics*.

[9] Matek Z., Jungvirth-Hegedus M., Kolacek S. (1999). Epidemiology of coeliac disease in children in one Croatian county: the cumulative incidence over ten-year period and the way of clinical presentation (part I). *Collegium Antropologicum*.

[10] Hegedus-Jungvirth M., Kolacek S., Zizic V. (2001). REzultati skrininga o prevalenciji celijakije u odrasloj populaciji u Međimurju. *Acta Medica Croatica*.

[11] West J., Logan R. F. A., Smith C. J., Hubbard R. B., Card T. R. (2004). Malignancy and mortality in people with coeliac disease: population based cohort study. *BMJ*.

[12] Green P. H. R., Fleischauer A. T., Bhagat G., Goyal R., Jabri B., Neugut A. I. (2003). Risk of malignancy in patients with celiac disease. *The American Journal of Medicine*.

[13] Husby S., Koletzko S., Korponay-Szabó I. R. (2012). European Society for Pediatric Gastroenterology, Hepatology, and Nutrition Guidelines for the Diagnosis of Coeliac Disease. *Journal of Pediatric Gastroenterology and Nutrition*.

[14] Greco L., Timpone L., Abkari A. (2011). Burden of celiac disease in the Mediterranean area. *World Journal of Gastroenterology*.

[15] Bonamico M., Nenna R., Montuori M. (2011). First Salivary Screening of Celiac Disease by Detection of Anti-transglutaminase Autoantibody Radioimmunoassay in 5000 Italian Primary School children. *Journal of Pediatric Gastroenterology and Nutrition*.

[16] Gatti S., Lionetti E., Balanzoni L. (2019). Increased prevalence of celiac disease in school-age children in Italy. *Clinical Gastroenterology and Hepatology*.

[17] Nenna R., Tiberti C., Petrarca L. (2013). The celiac iceberg: characterization of the disease in primary schoolchildren. *Journal of Pediatric Gastroenterology and Nutrition*.

[18] Comba A., Department of Pediatrics, Hitit University School of Medicine Training (2018). Prevalence of celiac disease among school-age children in Çorum, Turkey. *The Turkish Journal of Gastroenterology*.

[19] Raivio T., Kaukinen K., Nemes E. (2006). Self transglutaminase-based rapid coeliac disease antibody detection by a lateral flow method. *Alimentary Pharmacology & Therapeutics*.

[20] Costa S., Astarita L., Ben-Hariz M. (2014). A point-of-care test for facing the burden of undiagnosed celiac disease in the Mediterranean area: a pragmatic design study. *BMC Gastroenterology*.

[21] Fasano A. (2003). European and North American populations should be screened for coeliac disease. *Gut*.

[22] Mearin M. L., Ivarsson A., Dickey W. (2005). Coeliac disease: is it time for mass screening?. *Best Practice & Research. Clinical Gastroenterology*.

[23] *WHO Mass Screening Recommendations*.

[24] Ludvigsson J. F., Card T. R., Kaukinen K. (2015). Screening for celiac disease in the general population and in high-risk groups. *United European Gastroenterology Journal*.

[25] Catassi C., Gatti S., Fasano A. (2014). The new epidemiology of celiac disease. *Journal of Pediatric Gastroenterology and Nutrition*.

[26] Gomez J. C., Selvaggio G. S., Viola M. (2001). Prevalence of celiac disease in Argentina: screening of an adult population in the La Plata area. *The American Journal of Gastroenterology*.

[27] US Preventive Services Task Force, Bibbins-Domingo K., Grossman D. C. (2017). Screening for celiac disease: US Preventive Services Task Force recommendation statement. *JAMA*.

